# Wearable armband with a floating mobile exploratory electrode at fingertip for on-demand touch-and-measure multilead electrocardiography

**DOI:** 10.1017/wtc.2025.11

**Published:** 2025-05-05

**Authors:** Saygun Guler, Emre Aslanger, Murat Kaya Yapici

**Affiliations:** 1 Faculty of Engineering and Natural Sciences, Sabanci University, Istanbul, Türkiye; 2 Department of Cardiology, Basaksehir Pine and Sakura City Hospital, Health Sciences University, Istanbul, Türkiye; 3 (SUNUM) Nanotechnology Research and Application Center, Sabanci University, Istanbul, Türkiye; 4 Department of Electrical and Computer Engineering, University of Washington, Seattle, WA, USA

**Keywords:** electrocardiography, flexible bioelectrode, wearable graphene textile, smart medical garment, on-demand physiological monitoring, wilson common terminal

## Abstract

Spurred by the global pandemic, research in health monitoring has pivoted towards the development of smart garments, enabling long-term tracking of individuals’ cardiovascular health by continuously monitoring the electrocardiogram (ECG) and detecting any abnormality in the signal morphology. Many types of dry electrodes have been proposed as alternatives to gold standard Ag/AgCl wet electrodes, and they have been integrated into clothes capable of acquiring only a limited number of the different ECG traces. This limitation severely diminishes the diagnostic utility of the collected ECG data and obstructs the garment’s potential for clinical-level evaluation. Here, we demonstrate a special ECG upper armband with a glove component which houses graphene-textile electrodes, where a fully mobile, exploring electrode located at the index finger enables the user to strategically position the electrode on-demand to desired body areas and measure the different ECG traces that are bipolar limb and unipolar chest leads. Based on measurements with and without employing the well-known Wilson Central Terminal (WCT) arrangement, the correlation ratio of unipolar ECG chest leads acquired with the graphene textile-based armband and Ag/AgCl electrodes both in “WCT-less” configuration reach up to %99.65; and up to %99.54 when Ag/AgCl electrodes are utilized “with WCT” while the graphene-based armband in “WCT-less” configuration. To the authors’ best knowledge, this study reports the first multilead on-demand “touch-and-measure” ECG recording from a fully wearable textile garment. Moreover, owing to the human-centered armband design, we achieved a more than three-fold reduction in electrode count from 10 in clinical ECG practice down to 3.

## 1. Introduction

### 1.1. Background

An electrocardiogram (ECG) can provide a wide array of data on the functionality of a subject’s heart by measuring the electrical dynamics of the cardiovascular system from the skin surface (Sattar and Chhabra, 2022). The concept is based on the ability to detect the electrical activity using conductive patterns on the skin of a subject, which is then monitored as a voltage versus time graph. In a typical ECG setting, sticky Ag/AgCl electrodes are placed on the various parts of the body as biopotential sensors, and the potential difference is measured across several combinations of these electrodes. For the sake of research and simplicity, it is common to position one electrode on the left arm and another on the right arm, with an additional reference electrode placed anywhere on the body as “lead-I” configuration (i.e., *bipolar electrocardiography*); however, it is important to note that this configuration is very limited in its diagnostic utility within medical ECG framework (Trägårdh et al., 2006; Francis, 2016). In medical ECG, a thorough examination of the heart’s activity from multiple angles of view is performed by strategically positioning six electrodes on the chest and four on the limbs (Herring et al., 2018). This is achieved by creating a so-called zero-potential region (although this term has been the center of many controversies amongst the researchers who work on ECG over the years (Madias, 2008) on the frontal plane of the body, named the Wilson Central Terminal (WCT) (Einthoven et al., 1913). Later, this approach was improved and led to electronically more accurate configurations, thus creating *unipolar leads* in addition to the existing *bipolar leads* (Goldberger, 1942; Wilson et al., 1946) (The popular term “unipolar” used in this context is a misnomer as no configuration can actually form a single-pole potential difference). *The limb electrodes* consist of one on the left arm, one on the right arm, and one on the left leg. It is worth noting that there is also a reference electrode on the right leg; however, it is not utilized for measuring the potential difference but only for reducing noise artifacts and establishing a common ground (Gomez-Clapers et al., 2011). On the other hand, *the chest electrodes*, namely, V1–V6, consist of six electrodes placed along the fourth and fifth intercostal space (Rautaharju et al., 1998; Khunti, 2014). In a short summary, a standard ECG sheet typically comprises 12 traces, out of which, six are derived from the chest by referencing to the WCT and called as the unipolar chest leads (V1–V6), three are obtained from the limbs and named as the bipolar limb leads (lead I, lead II, and lead III), and the remaining three again are derived from the limbs by referencing to a modified WCT and termed as the augmenented unipolar limb leads (aVF, aVR, aVL).

### 1.2. Literature review

With the increasing rate of cardiovascular diseases around the globe (Timmis et al., 2022), wearable technology has evolved from basic activity trackers, such as smartwatches and bracelets (Wu et al., 2016), to high-grade medical products that can be worn for prolonged periods with utmost reliability and patient comfort (Dagher et al., 2020; Smuck et al., 2021). The diagnostic accuracy and practicality of long-term out-of-hospital-ECG have previously demonstrated superiority compared to instant clinical-ECG assessments in several cases (Ioannidis et al., 2001; Graatsma et al., 2009); therefore, it is crucial to ensure convenient clinical measurements in a home setting, especially for vulnerable groups like the elderly and infants, as it can yield significant advantages in managing cardiovascular diseases.

Conventional sensors used in medical ECG procedures are commonly Ag/AgCl wet electrodes (Liu et al., 2015). Although they have proved to be highly useful for instant measurements, many studies have reported adverse effects of this type of disposable electrodes when they are stuck onto the skin for an extended period of time, which is unsuitable for long-term measurements (McNichol et al., 2013; Fumarola et al., 2020). These limitations prompted researchers to develop dry, soft, comfortable, and innovative alternatives to wet electrodes, enabling subjects to continuously wear them for extended periods, potentially spanning days or even weeks. Dry electrodes are suitable for continuous cardiovascular health tracking because they do not require time-consuming skin preparation with viscous gels as wet electrodes do, and they even conform very well to rough skin surfaces with their flexible structure (Meziane et al., 2013).

In tandem with advancements in flexible electronics, which have led to thinner and softer wires along with smaller electronic circuit boards, many attempts have been made to enhance the quality of such biopotential sensors, refine their production techniques, and facilitate their ease of wearability, experiencing notable acceleration, particularly since the 2000s. Shirt-like garments were proposed where a limited number of ECG tracks can be realized using stainless steel (Bourdon et al., 2005) as well as chest bands that can host a very limited number of ECG leads (Shen et al., 2006). Various knitting techniques were assessed based on the quality of the ECG signals they yielded (Fobelets et al., 2023). Several types of bras containing textile electrodes were developed as woman-specific garments that are capable of measuring three-lead ECG (Schauss et al., 2022). A smartwatch design was proposed with the capability to measure ECG on the Einthoven triangle; however, it can only realize Wilson leads when it comes into contact with the electrode locations on the body, rather than while being worn constantly on the wrist (Samol et al., 2019). 12-lead ECG was successfully realized on an upper body garment and benchmarked against a gold-standard Holter ECG device (Fouassier et al., 2020). Further, specialized garments containing textile electrodes were designed for specific professional groups such as cyclists (Paiva et al., 2019).

However, these earlier attempts included cumbersome electronic boards and sewing techniques with (mostly silver-based) conductive coatings on fabrics (Ottenbacher et al., 2004). Silver was later proposed to be printed on thermoplastic polyurethane (TPU) substrate layer for a monitoring garment, and it was tested in several activity scenarios such as walking and climbing while being worn (Bu et al., 2021). Nylon threads were coated in silver, and two-dimensional t-shirt designs along with three-dimensional simulations were realized as health-tracking garments using industrial-level software such as Marvelous Designer (Malek et al., 2022). Tattoo electrodes were proposed for around-the-ear ECG measurements using inks that contain silver nanoparticles (Jacob et al., 2018). A chest band type was proposed that contains conductive yarn with silver-treated nylon partially covering the abdomen region when worn (Abtahi et al., 2015). Hydrophilic polyurethane- and silver-based paste was proposed to increase the contact stability between the skin and electrodes and to decrease the skin–electrode impedance (Soroudi et al., 2019). Inkjet-printable silver inks were printed on a polyimide substrate (Momota and Morshed, 2020). Carbon-derived materials have also been extensively used in biopotential measurements such as reduced graphene oxide (rGO) due to their exceptional electrical and mechanical properties, making it an ideal candidate for the wearable smart garment concept (Yapici and Alkhidir, 2017; Mirbakht et al., 2023). Carbon paste was proposed to make conformable skin patches as electrodes when left dry on the skin for several minutes (Lee and Yun, 2017). Silver nano-wires combined with GO were printed on a PET substrate (Xu et al., 2019). Since graphene is primarily available in water-based solutions, textiles were made dip-coated with graphene to gradually achieve the desired level of conductivity through multiple coating cycles (Yapici et al., 2015). Vacuum filtration was used to deposit GO suspension in a polyester fiber (Lou et al., 2016). CO_2_ laser production techniques have been used for graphene to be printed on a PDMS substrate as an ECG electrode (Yang et al., 2021). Further, graphene not only found relevance in wearables but also captured considerable interest in hearables, particularly in products designed to track health information from around the ears (Guler et al., 2022). Spray printing was one of the feasible techniques used with graphene material for making single-arm textile ECG electrodes (Ozturk et al., 2023). Chemical vapor deposition (CVP) was exploited to coat graphene on a silver chloride-based substrate (Celik et al., 2016). Stencil printing was used with carbon-loaded rubber for making active electrodes and benchmarked against commercial-level passive ECG sensors (Paul et al., 2015). Polymers, on the other hand, have garnered significant attention as potential building blocks due to their thermal and chemical stability such as PEDOT:PSS (Pani et al., 2016). Polyester nanofiber yarns coated with PEDOT:PSS were proposed, and their durability tests revealed promising results (Tsukada et al., 2019). Though not wearable, PEDOT:PSS was printed on a paper for making ECG electrodes and achieved high ECG signal quality (Bihar et al., 2017). Polyester-based electrodes, resistant to humidity and high temperatures, were developed (Wang et al., 2022). Polymer-supported biocompatible stand-alone electrodes were proposed, and they offered a degree of flexibility that is feasible for integration into regular everyday clothes (Hoffmann and Ruff, 2007).

Graphene stands out among many materials used for ECG electrode making due to its exceptional properties, such as thermal and electrical conductivity (Balandin et al., 2008; Cao et al., 2015), durability (Abbas and Hussein, 2022), and dimension (Rao et al., 2009). In this work, we harnessed the intrinsic qualities of graphene and proposed an innovative armband design tailored for capturing multichannel ECG measurements from the wearer. This sophisticated armband includes a glove component seamlessly connected to the biceps region via a textile piece that extends to cover a part of the elbow and lower arm. At the heart of this design is a unique feature: an exploring electrode sewn at the fingertip of the index finger on the glove. This electrode is fully mobile, giving the wearer the freedom to position it anywhere on their body within reach. This flexibility enables ECG measurements to be taken from multiple angles (that is leads) and various locations on the body, unlike everyday smart devices such as smartwatches, which can only record ECGs from a single angle; typically from a wrist or an arm. The multilead measurement capability is therefore critical for capturing the entire footprint of the clinically relevant cardiac activity to enable meaningful medical diagnostics.

## 2. Methodology

**Figure 1.**
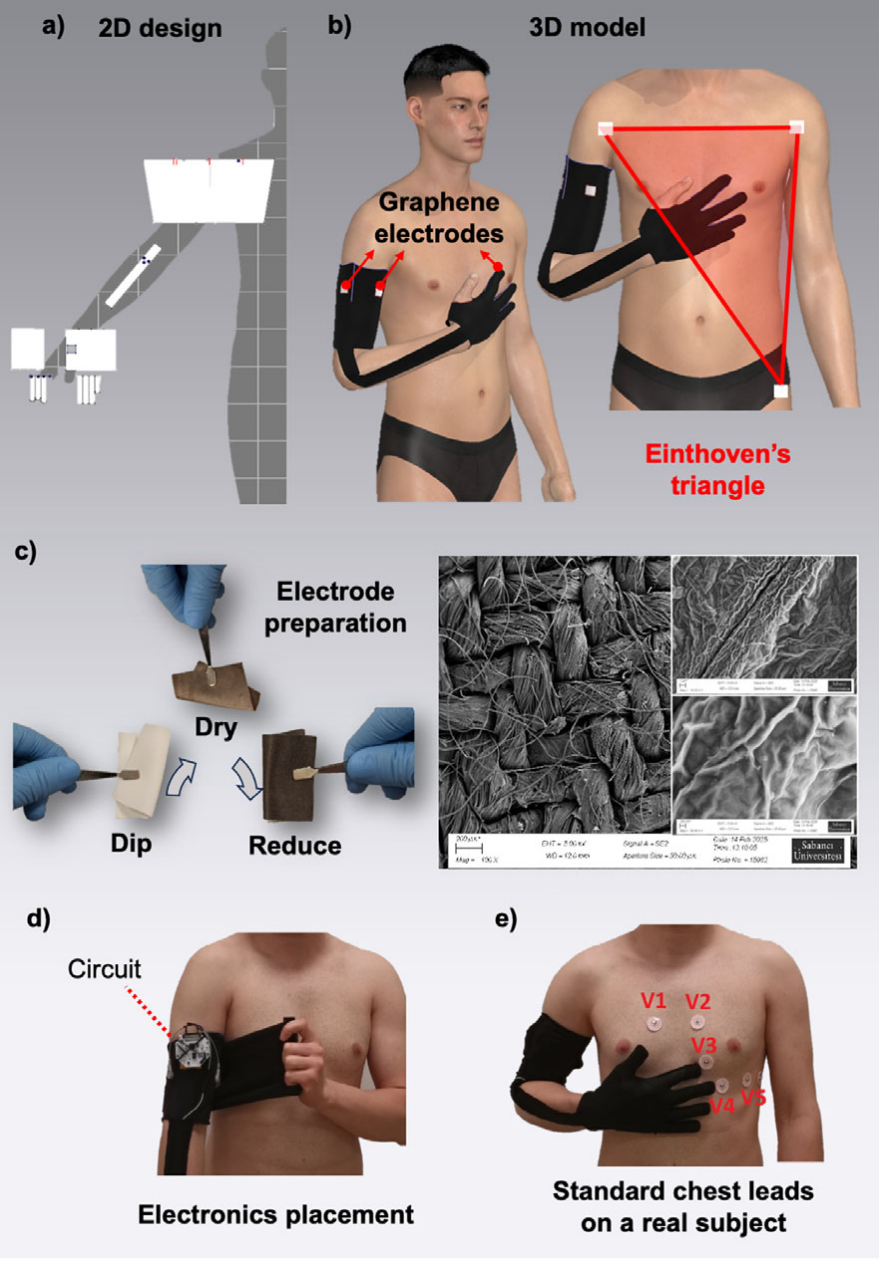
Visual abstract of the study: (a) 2D armband design: This is prepared in Clo3D software before simulating it on a human avatar. (b) 3D model of the armband: The simulation was performed using the same software tools, and one of the default human avatars was used. Graphene electrodes were integrated, with two positioned within the upper arm component and one situated at the fingertip of the index finger within the glove. (c) A feasible, fast, and inexpensive dip-dry-reduce technique: Bamboo nylon textile was coated with graphene oxide solution, followed by a reduction process to enhance the electrodes’ conductivity. Scanning electron microscope (SEM) images reveal the surface morphology of graphene textile electrodes. (d) The armband was crafted using a dual-layer design, concealing the electronic components within. This construction ensures comfort and aesthetics in that when viewed from the outside, no unsightly wires or circuits are visible. (e) Standard ECG chest leads V1 to V6 on a real subject: The armband was tested in every combination of a traditional medical ECG sheet.

The fundamental notion behind this research was to realize medical-level multichannel ECG using a minimum number of sensing bits with maximum feasibility and comfort on a health-tracking fashionable cloth for everyday use. Theoretically, not every lead in ECG is measured independently. For example, augmented limb leads can be computed mathematically, and leads I, II, and III follow a triangular vector sum relationship (Macfarlane et al., 2010). Thus, the total number of electrodes on the armband could be reduced to three: two on the upper arm and one on the fingertip of the index finger. As shown in Figure 1a, the two-dimensional blueprint of the product was first designed in a commercial fashion design software, Clo3D (© CLO Virtual Fashion LLC). The upper arm component featured a trapezoid shape, whereas the glove part was fashioned using a variety of rectangular shapes, including the middle bridge, which connects the upper arm component to the glove. To enhance the aesthetic appeal, several *curvature points* have been incorporated at the edges of the fingertips. The electrodes were drawn on as internal rectangles and cloned precisely on top of the cut shapes in 2D panel of Clo3D, and an additional fabric was marked in a contrasting color to visually distinguish the electrodes from the default garment texture. While the particle distance of the finger components within the glove was set to 5 mm due to their small size, the particle distance of the upper arm component of the armband was set to 10 mm. *‘Tack on avatar’* command under *‘Sewing’* menu toolbar was used to fixate the simulated textile at two specific locations: directly below the elbow and at the top of the upper arm. The textile pieces were merged together using only *segment sewing* feature. One of the default human avatars was used, and the posture was adjusted using *‘Show X-ray Joints’* feature. No additional alterations were made to the 3D physical model such as hair, size, shoes, or texture.

The armband featured a sleek design, crafted from durable black cotton fabric. The assembly of the fabric components into the final product was meticulously performed using Promise 1408 sewing machine (Singer Sourcing Limited LLC). The electrodes of the product were prepared using bamboo nylon textile, carefully coated with reduced graphene, and seamlessly integrated into the cotton fabric during the product’s final assembly. The locations of the three conductive textile pieces were depicted in Figure 1b. These electrodes were prepared in a three-step process as graphically summarized in Figure 1c. Surface texture, tightness, and thickness play pivotal roles in textile coating, and careful control of these parameters is crucial throughout the process. Extensive testing has been conducted on a range of textiles, including bamboo nylon, polyester, and cotton, to achieve the most favorable deposition results. Ultimately, due to its notably low surface roughness, which results in a uniformly applied graphene oxide (GO) coating, nylon stands out as the preferred plain textile choice for ensuring optimal coating uniformity. A large piece of bamboo nylon textile was coated with a water-based GO solution with 4 mg/ml concentration prepared via the modified Hummers’ method (Hummers and Offeman, 1958). After the coated fabric was left to desiccate on a hot plate at 80 °C, it was dipped in and reduced using sodium borohydride solution ( 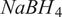 ). While thermal reduction was a potential method, it is essential to consider the risk of textile disintegration at elevated temperatures. Consequently, the approach of coating with a GO solution followed by its chemical reduction was a more attractive and viable alternative, and thus selected. After the reduction process, the large piece of textile was immersed in deionized (DI) water for cleansing. This step was for removing chemical residues from the fiber surface and ensuring that only rGO flakes remained adhered to the textile. The product was then carefully placed on a hot plate in a glass container to air-dry for a sufficient amount of time to get rid of excess damp. Throughout the reduction of GO, a noticeable transformation occurred in the color of the textiles. They transitioned from a brown hue to a deep black shade. This color shift was a direct consequence of the removal of oxygen-containing groups from the material’s surface, and it was accompanied by a significant enhancement in electrical conductivity. Thus, the alteration in textile color serves as a valuable qualitative indicator of the reduction process’s effectiveness; while quantitatively, the reduction was also verified by monitoring the resistance of the textile piece. After achieving the desired surface resistance on the large textile, which was approximately 1 k 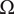 on 1 cm-long fabric, small pieces were meticulously cut to prepare the graphene textile electrodes for integration into the armband. The textiles were carefully cut into pieces measuring 1.5 cm by 2 cm using scissors. Rubber isolation tapes were affixed using a commercial textile adhesive just beneath the conductive textiles to establish a sturdy support and ensure the necessary pressure when in contact with the skin. Snap fasteners were subsequently incorporated, securing the textile electrodes in place. Thin metal wires, with a diameter of 250 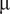 m, were soldered to establish the connection with the metal fasteners. In their final form, the electrodes possess a standalone shape and can be replaced if damaged, while also being mountable to the designated locations on the armband. The methodology used for fabricating the graphene textile electrodes in this study follows on from our previous works, which also include comprehensive material characterization results (Yapici et al., 2015; Ozturk et al., 2023). Although the electrodes were fabricated using the previous approach, the glove component demanded a much sturdier wiring process due to the significantly greater distance between the index finger electrode and the location of the electronic board. This extended span increases the risk of motion-induced noise. Unlike previous studies, where electronic boards were typically placed near fixed armband electrodes – restricting signal acquisition to predetermined body regions – the glove design allows the wearer to capture signals from any location they can touch, greatly enhancing flexibility and usability.

The armband’s upper arm component consists of a two-layer design as shown in Figure 1d. The first layer, in direct contact with the skin, houses two electrodes: the reference electrode and the first differential electrode (The second differential electrode, enabling potential difference measurements, was positioned at the tip of the index finger). The second layer, the outermost one, functions as a protective cover. This design incorporates the biopotential circuit between the two layers, with the upper layer serving to conceal this circuit for aesthetic purposes. This clever construction ensures that when observed from the outside, no wires or circuits are visible, creating a sleek and seamless appearance. The biopotential circuit used in the armband was an open-source data acquisition unit equipped with no extra mechanical amplification and filtering (Cyton Board, OpenBCI). The raw data collected from the board were first stored in the local server as .txt files and then processed using custom-written MATLAB scripts (MathWorks, Natick, Massachusetts, USA).

The fingertip electrode was integrated during the assembly of the armband’s glove component. To achieve this, the fingertip was horizontally cut, allowing for the insertion of a metal fastener beneath the outer layer. An additional thin fabric layer was skillfully sewn beneath this outer layer to ensure that the wearer would not experience any discomfort from the wiring. This additional layer extended from the fingertip all the way to the electronic circuits. It is worth noting that electrical interconnects are a typical challenge for all types of electrodes. Numerous research efforts have been dedicated to finding the optimal method for networking and integrating conductive hard metals or materials with soft conductive textiles (Paul et al., 2014). This includes developing reliable connections to the electronics board and ensuring the entire system is motion-robust while minimizing artifacts. With the methodology we present in this work, we were able to securely interface the readout electronics with the textile electrodes housed inside the armband and glove units.

## 3. Results

### 3.1. Benchmarking of the textile electrodes

To assess the performance of the newly developed dry textile electrodes and the accompanying armband, a series of tests were conducted on three healthy participants with no prior history of cardiovascular disease. Initially, the graphene electrodes were benchmarked against the gold standard Ag/AgCl electrodes as a side-by-side evaluation. This comparison involved acquiring simultaneous ECG signals in the lead-I configuration. Both electrode types were affixed to the volunteer’s right and left arms, with a common reference point established using a graphene electrode. The ECG data was recorded using a Cyton Board, OpenBCI, utilizing its first two-channel pins. The signal qualities were quantitatively measured as signal-to-noise ratio (SNR) in dB unit. The total energy surrounding QRS complex was defined as the signal, and fluctuations surrounding the region that’s between consecutive S- and Q-containing P-, T-waves, and isopotential lines were defined as noise (Equation 3.1). The same rule was applied when also making the RMS noise analysis.(3.1)

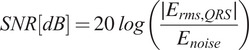



Several filtering steps were performed offline using MATLAB scripts. The initial step involved the elimination of powerline interference noise at both 50 and 100 Hz from the ECG signals that had been normalized. This was achieved by applying a notch filter with a Q factor of 35. Subsequently, a bandpass filter was implemented to effectively attenuate noise components that were either below 5 Hz or above 50 Hz. To enhance the signal’s overall quality and smooth it, a fifth-order moving average filter was then employed.

**Figure 2.**
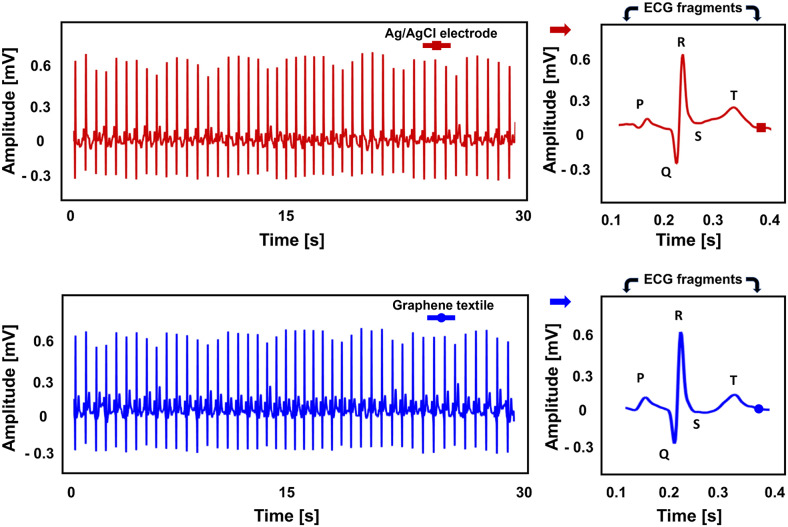
Two simultaneous ECG recordings were acquired from the same participant, with sensors placed adjacently on the left and right arms in lead-I configuration. While Ag/AgCl electrodes provide high-quality results in almost every case for instant measurements, graphene textile electrodes exhibited slightly superior signal-to-noise ratio (SNR) scores, demonstrating improvements of up to 1.2 dB.

Figure 2 shows the signals taken with Ag/AgCl and graphene textile electrodes with participant #1, respectively. The Ag/AgCl electrodes yielded an SNR of 14.76 dB with RMS noise of 75.82 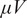 , while the graphene textile electrodes exhibited a slightly superior SNR of 15.91 dB with less RMS noise of 67.93 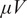 . Additionally, the correlation ratio of these two signals was %99.6, and mean root sum of squares (RSS) around QRS complex (signal) for commercial and graphene electrodes are 1.72 and 1.67 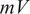 , and mean RSS between QRS complex (noise) are 0.71 and 0.69 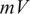 , respectively. It is worth noting that even though the marginal dB difference could be attributed to minimal variations in electrode placement, likely within mere millimeters, these results explicitly highlight the potential of graphene textile electrodes as exceptional alternatives to the gold-standard Ag/AgCl electrodes.

As mentioned in the methodology section, the graphene textile electrodes incorporated into this armband were originally developed and characterized in our previous studies (Yapici et al., 2015; Ozturk et al., 2023). Since the same techniques were used, the empirical tests conducted during sitting and walking yielded similar results. These results confirm that our graphene textile electrodes remain highly functional and usable even for long-term measurements. However, this multichannel armband is not specifically designed for uninterrupted use such as a Holter monitor with recording durations extending up to 48 h; but rather, for situations where the wearer needs to record multichannel ECG by briefly holding their touch on the relevant region of interest on the chest or other areas to acquire meaningful ECG signals to be transmitted for remote, on-the-spot telehealth assessment of their cardiac condition based on the clinically relevant bipolar ECG limb and unipolar chest leads. Our tests demonstrate that even when the participant is walking, the signal quality does not degrade as long as the wearer maintains the finger contact (the electrode on the glove) with the measurement location on the skin surface, indicating that the system as a whole is resistant to motion artifacts. The noise generated by walking motion can be effectively suppressed using passive filters, and the increase in high-frequency noise is limited to approximately 5–6 dB, not only for graphene textile electrodes but also for commercial adhesive Ag/Cl electrodes. This suggests that graphene textile electrodes have significant potential as wearable alternatives for biopotential monitoring.

### 3.2. Skin–electrode impedance

Skin–electrode impedance serves as a pivotal quality metric for biopotential electrodes, where lower and consistently stable impedance values are indicative of enhanced performance. This critical relationship has been electronically modeled and rigorously examined through experimentation, employing LCR meters, as extensively detailed in (Gan et al., 2022) where 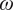 is the angular frequency (rad/s), *Q* is the capacitive effect of the electrical behaviour of the epidermis ( 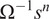 ), n is the constant representing inhomogeneities of the interface, and finally Z is the impedance (Equation 3.2).(3.2)

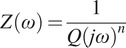



**Figure 3.**
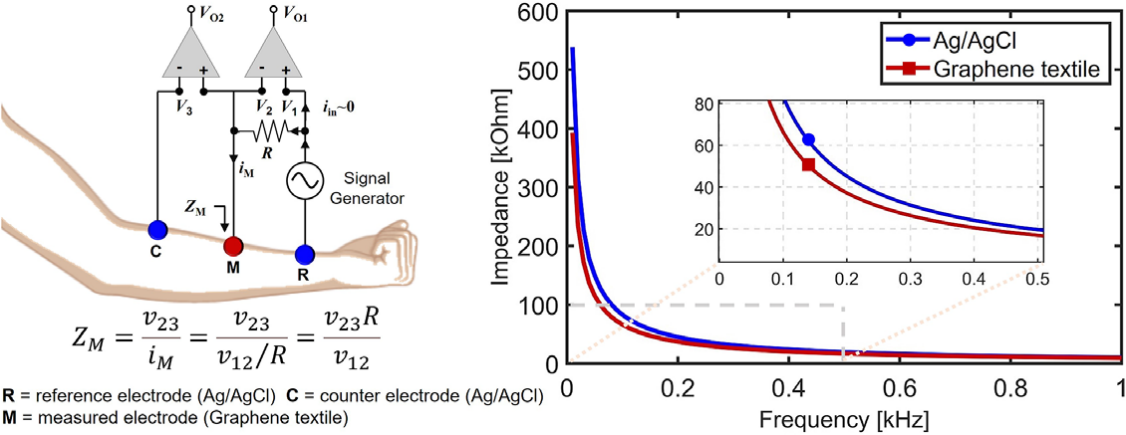
Skin–electrode impedance of the graphene textile electrodes and commercial wet Ag/AgCl electrodes. While the conventional Ag/AgCl electrodes show between 80 and 32 k 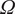 impedance, printed electrodes show 65–26 k 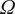 between the frequency range of 0.1–0.3 kHz.

The impedance of graphene textile electrodes was compared to that of commercial Ag/AgCl electrodes. An impedance measurement circuit, utilizing a conventional Howland current pump, as previously described in studies (Spach et al., 1966; Yapici et al., 2015), was constructed to assess and contrast the impedance characteristics of these two types. The setup is shown in Figure 3. A sinusoidal current was introduced into the skin of the participant, gradually varying in frequency from 1 Hz to 1 kHz, at intervals of 5 seconds in a sweeping trend. By taking the ratio of the voltage difference between the target and counter electrodes to the current from the reference electrode, the impedance of the electrode–skin interface was calculated. The equation governing the impedance of the skin–electrode interface is presented in Figure 3 where 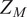 is the contact impedance between the skin and the measured electrode (M), 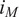 is the current going into the measured electrode, 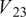 is the voltage drop on 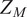 , and 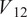 is the voltage drop on the resistance R.

In both experimental phases, impedance measurements were conducted on two distinct types of electrodes, with the first phase involving commercial electrodes and the second phase featuring graphene electrodes. These were positioned on the first participant’s forearm using sturdy tape, 5 cm away from each other. Figure 3 shows the impedance results. While the conventional Ag/AgCl electrodes show between 80 and 32 k 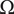 impedance, printed electrodes show 65 and 26 k 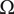 between the frequency range of 0.1–0.3 kHz. These findings clearly corroborate the earlier results, wherein SNR values were nearly identical when measured with both Ag/AgCl and graphene textile electrodes.

### 3.3. Performance tests of the armband

One of the biggest challenges in the wearables industry revolves around the limitations (or sometimes even restrictions) surrounding the acquisition of diagnostic information (Walker et al., 2016; Kumar et al., 2023). Wearable health products often fall short in providing comprehensive, intricate data for accurate diagnosis. Medical facilities, like hospitals, on the other hand, employ an extensive array of cutting-edge tools and devices that offer a holistic and in-depth perspective when examining a subject’s health, affording healthcare professionals the ability to assess patients from a multifaceted standpoint, thus allowing for more precise diagnosis and treatment. A notable illustration of this difference lies in the capabilities of armbands, bracelets, and smartwatches, which are limited to measuring a single channel of ECG (Ozturk et al., 2023), or photoplethysmogram (PPG) (Guler et al., 2023). In contrast, Holter devices or conventional multichannel ECG tools equipped with Ag/AgCl electrodes offer a far more comprehensive array of data, capturing up to 12 distinct (yet dependent) traces.

The armband developed and proposed in this study was rigorously tested to assess its capabilities, not only for surface-level heart rate measurements in a single-ECG trace but also, and more importantly, for advanced 12-lead ECG trace capabilities using a biopotential measurement circuit, Cyton Board, OpenBCI. To do this, two sets of experiments were conducted, one without the WCT setup (i.e. “WCT-less”) and one with the WCT.

In the first case, both the Ag/AgCl electrodes and the graphene-based armband were tested in the “WCT-less” configuration, to evaluate the performance similarity of electrodes in recording the unipolar chest leads. Three participants were recruited aged 26, 31, and 32. They were seated in an enclosed, isolated room with no external influences, such as natural light, noise, or any other physical disturbances. Initially, two Ag/AgCl electrodes were affixed to participants’ right upper arm. Subsequently, they wore the graphene armband developed for the experiment. The armband was slightly adjusted to position the graphene electrodes in very close proximity to the AgCl electrodes that had been previously affixed. The commercial chest electrodes were positioned as shown in Figure 1e. During the data collection process, the participant was instructed to place their index finger next to each wet electrode in every cycle. Data were collected at 2-minute intervals, with each data collection session lasting approximately 3 minutes. This duration excludes the time spent on transitions between electrode combinations, which involves instructing the participant, electrode placement, and data preprocessing on the computer interface. The processing steps applied to the collected signals remained consistent with those employed in the previous experiment. Likewise, the same passive bandpass and notch filters were used with previous settings.

Table 1 displays an analysis and comparison of signals obtained simultaneously from both the developed armband and commercial Ag/AgCl electrodes across all chest lead combinations, from V1 to V6 for participant #1. It is important to note that, for ease of ECG data interpretation, only the signal analysis data from the first participant is presented in this manuscript. However, data from participants two and three, which exhibit strong consistency with the first participant’s data, are included in the Supplementary Materials. In each instance of the first participant, the correlation ratio consistently exceeds %94, underscoring the significant accuracy and exceptional quality of the graphene electrodes in capturing ECG readings, rivaling the performance of commercial-grade electrodes. Notably, the highest correlation ratio is %99.65 in V6, while even the lowest, in V4, maintains %94.64. These ratio scores demonstrate that the pressure exerted on textile electrodes of the armband applied to the skin is nearly on par with the adhesive capabilities of commercial electrodes, showcasing their comparable performance. The SNR scores also exhibit a harmonious variation across different leads. In V3, both commercial and graphene electrodes have the highest SNR scores of 24.63 and 26.84 dB, respectively. The most significant difference between the two types was observed in V4, with a 2.7 dB. In this case, the SNR for commercial electrodes stands at 3.94, whereas the armband has an SNR of 1.24 dB.

**Table 1.** Results for participant #1: Signal analysis was conducted on the graphene textile armband and compared with commercial Ag/AgCl wet electrodes

Feature 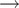 Configuration 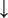		Correlation ratio	SNR score [dB]	RMS noise 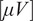	Mean RSS around QRS complexes 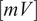	Mean RSS between QRS complexes (noise) 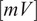
V1	Graphene textile	% 96.28	5.87	18.15	1.11	0.19
	Ag/AgCl		8.53	13.47	1.17	0.14
V2	Graphene textile	% 96.38	18.04	64.24	1.94	0.68
	Ag/AgCl		20.06	42.61	1.74	0.45
V3	Graphene textile	% 99.62	26.84	79.46	3.6	0.78
	Ag/AgCl		24.63	87.3	3.52	0.86
V4	Graphene textile	% 94.64	3.94	277.96	1.54	2.17
	Ag/AgCl		1.24	282.65	2.11	2.2
V5	Graphene textile	% 99.01	5.8	254.64	2.88	2.06
	Ag/AgCl		7.2	233.87	3.01	1.9
V6	Graphene textile	% 99.65	13.91	130.23	2.47	1.11
	Ag/AgCl		12.47	149.59	2.62	1.27

*Note.* This table shows the assessment of signal quality characteristics in the absence of a Wilson Central Terminal (WCT) setup, employing only three electrodes for each electrode type: two differentials and one reference.

In the second and third participants, the correlation ratio drops to 81% for the V1 configuration alone. However, this is still considered a high correlation. It is important to note that when participants contract their biceps, high-frequency noise increases, often necessitating adjustments to the filter parameters. For the sake of comparison, however, we maintained stable passive filters throughout the analysis. Despite the drop in correlation ratio to 81%, the signal power of both commercial electrodes and graphene armbands remains strongly correlated – when one decreases, the other also decreases, and when one increases, the other follows suit. The SNR scores range from 7.83 to 17.65 for participant 2 and from −0.06 to 9.8 for participant 3. Meanwhile, Figure 5a illustrates the interrelationship between the two types of electrodes concerning their quantitative signal quality.

It is worth noting that the signal quality indicator used here is a high-frequency noise-centric measure and should not be misinterpreted. Since it calculates the ratio of QRS segment energy to isopotential line energy, the output score standard may vary between different configurations and for experiments performed at different time intervals. Because the QRS amplitude is taken into account, even a slight electrode displacement of just a few millimeters in a second measurement; or a measurement that it is performed after a certain time where fluctuations could have occurred either in the skin conditions (due to sweat, humidity etc.) or in the persons’ physiology, can eventually lead to variations in the output SNR score. For this reason, the metric should be used to compare electrode types, such as graphene textiles versus commercial wet electrodes, in experiments that are performed simultaneously or sequentially with a minor time delay; rather than to compare different configurations or trials that were performed with hours of gap in time. In simple terms, this metric is for direct comparison between electrode types tested ideally at or near the same timestamp to properly subject the different electrode types to similar physiological and skin–electrode conditions, as well as, the ambient noise sources. Even small variations, such as shifting the electrode position by a few millimeters, changing the participant, or selecting a different ECG fragment within the region of interest, can significantly impact the output SNR score.

The highest RMS noise in 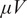 was notably detected in leads V4 and V5, which aligns with the expected pattern due to slightly lower SNR in these leads compared to others. Specifically, the maximum RMS noise was registered at 282.65 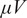 for conventional commercial electrodes, while the graphene textile armband exhibited a slightly lower value of 277.96 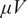 , indicating a marginal difference of 4.96 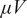 . Additionally, around the QRS complex, the mean “root sum of squares” (RSS) attains its peak, with values reaching approximately 3.6 mV for the armband electrodes and 3.52 mV for the commercial electrodes, notably observed in lead V3. Furthermore, the mean RSS between these QRS complexes is most pronounced in lead V4, registering at 2.17 mV for the armband electrodes and 2.2 mV for the commercial electrodes.

The second phase of the study involves a setup “with WCT” arrangement for Ag/AgCl electrodes and “WCT-less” configuration for the graphene textile-based armband. This approach seeks to assess and compare the medical-grade capabilities of the newly developed armband for on-demand “*touch-and-measure*” multilead ECG and perform a morphological analysis of two signal sets, contrasting their distinct signal and noise components. WCT is an artificial reference point system where the potential difference from the electrodes was measured across as unipolar leads, and it has been the center of many disputes amongst medical researchers recently. Although widely employed in nearly all medical ECG devices, there is a prevailing viewpoint that the concept of WCT may not establish a truly cohesive triangular for creating well-defined reference point centered around the heart (Gargiulo et al., 2018). To determine the veracity of this phenomenon and evaluate its validity, distinct equipment configurations and experimental setups involving many participants is needed. However, in our study, we have opted to conduct a more direct approach and make a comparative analysis of signal morphology, directly contrasting data obtained using the WCT-equipped setup containing commercial wet electrodes, with that obtained from an armband devoid of WCT arrangement (“WCT-less”) that possesses only three embedded graphene textile electrodes. The WCT configuration was implemented using a breadboard and 5k 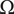 resistors. Each component of the WCT triangle was routed to the breadboard via metal and shielded wires, and then connected to a 5k 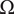 resistor. From there, the signal was directed to the appropriate pins of the Cyton, OpenBCI, a biopotential measurement board available for data acquisition. The environment was exactly the same as the first phase of the study, where the participant sat on a stable chair in an isolated room shielded from external influences. The signals were again filtered offline in MATLAB, and the data were collected at 2-minute intervals, with each data collection session lasting 3 minutes. A total of ten Ag/AgCl electrodes were securely affixed on the participant’s body. Three of these electrodes were utilized to establish a WCT: one on the right arm, one on the left arm, and one on the left leg. Additionally, six electrodes were placed strategically on the chest, as shown in Figure 1e, and one electrode was designated as the reference electrode of the entire electronics on the right leg. Subsequently, the participant was instructed to don the newly developed textile armband.

**Figure 4.**
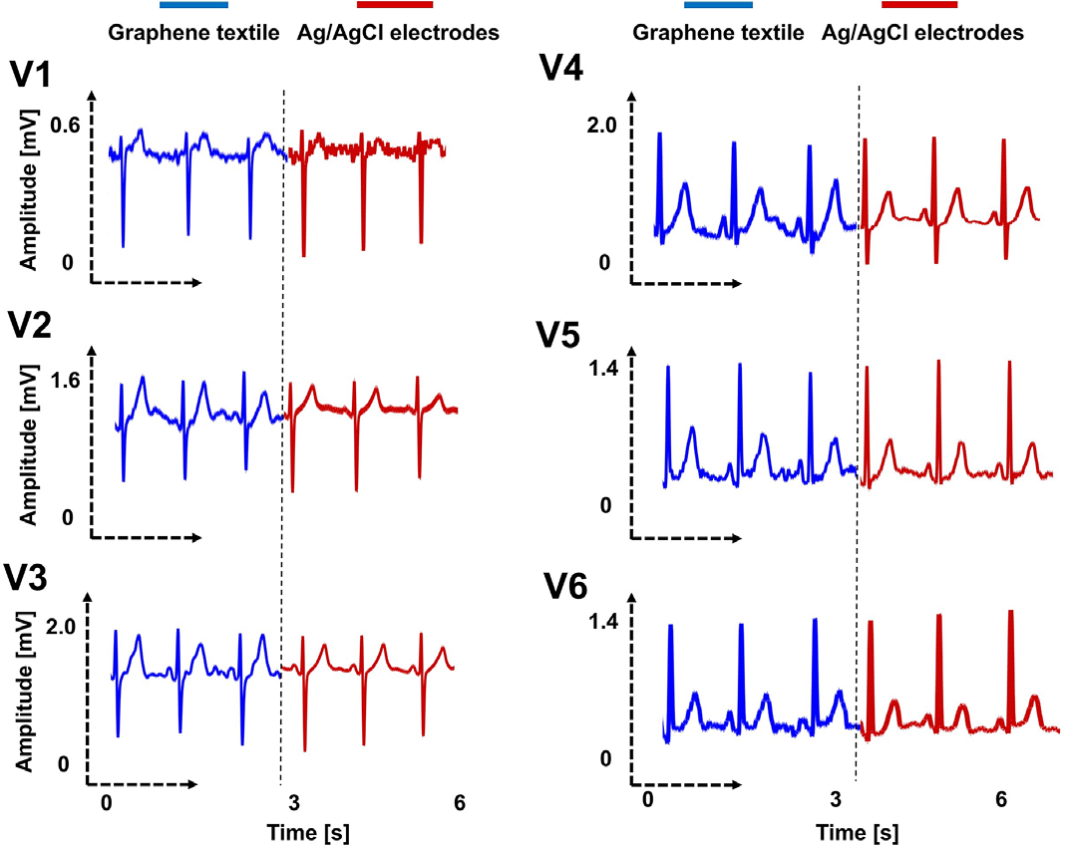
Unipolar chest leads were taken using the newly developed textile armband constructed with graphene electrodes. Simultaneously, ECG data were recorded using Ag/AgCl electrodes in a separate channel. Correlation coefficients were computed and reported in Table 2, revealing a significant level of similarity between the two datasets. Furthermore, the signal-to-noise ratio (SNR) values were found to be remarkably close. The blue trace illustrates the data obtained from the graphene armband without the application of the Wilson Central Terminal (WCT) setup, while the red trace represents the data collected with the Ag/AgCl electrodes and WCT setup in place. These results rekindle the ongoing debate regarding whether WCT is indeed an indispensable requirement in all scenarios.

Figure 4 shows the synchronously collected unipolar chest leads traces using the textile armband with three graphene electrodes and ten commercial Ag/AgCl electrodes for participant #2. To clarify, the positions of the Ag/AgCl electrodes are shown in Figure 1e. Participants wearing the designed armband, which includes the glove component, were instructed to touch the areas on their body directly beneath the corresponding chest leads. Specifically, their index fingers were positioned to slightly touch the adhesive plastic surrounding the commercial electrodes. This setup was intended to minimize the distance between the two types of electrodes during benchmarking. Upon initial inspection, a striking similarity emerges between the ECG signals obtained from the armband and those recorded using wet electrodes with WCT arrangement. Externally, they appear to exhibit a certain degree of harmony. We note that in a typical, healthy ECG morphology, the nature of ventricular depolarization causes the ratio of QRS amplitude to T-wave amplitude to shift from negative to positive as one moves from lead V1 to V6 (Herring et al., 2018). This transition is clearly observable in the recorded unipolar ECG chest leads (Figure 4). However, when we delve into their morphological characteristics, small differences may begin to surface. The most conspicuous difference lies within the first signal V1, the one represented by the blue trace, which originates from the armband. In comparing these two ECG traces, it is worth noting that while there is some similarity in the morphology of the T waves, it is not particularly pronounced. The shape and sharpness of the peaks of these waves appear somewhat distinct when contrasted with other V2–6 traces. We attribute this phenomenon primarily to the participant maintaining their arm in a contracted position for an extended period of time, which keeps the biceps taut while placing a finger electrode on the V1 location, situated on the right side of the chest plane. This enforced muscular activity generates electrical signals known as electromyography (EMG) (Ozturk et al., 2021; Ozturk and Yapici, 2021), which have the effect of attenuating or suppressing the ECG signals on these pivotal segments of the recordings. The second notable aspect of this graph is the difference in T-wave amplitudes for V3 and V4 configurations. Graphene electrodes clearly display elevated T-wave peaks in both instances. This discrepancy can be ascribed to the challenge of obtaining simultaneous recordings for a fair morphological comparison. The positioning of the electrodes is not always precisely in anatomically correct locations but rather in close proximity to their alternatives. For instance, it may not always be feasible to position two distinct types of electrodes precisely between the fourth and fifth intercostal spaces due to limited adjacent space. Consequently, electrode placement in this phase of the study involved a degree of arbitrary decision-making, requiring a discerning eye for the most suitable location.

**Figure 5.**
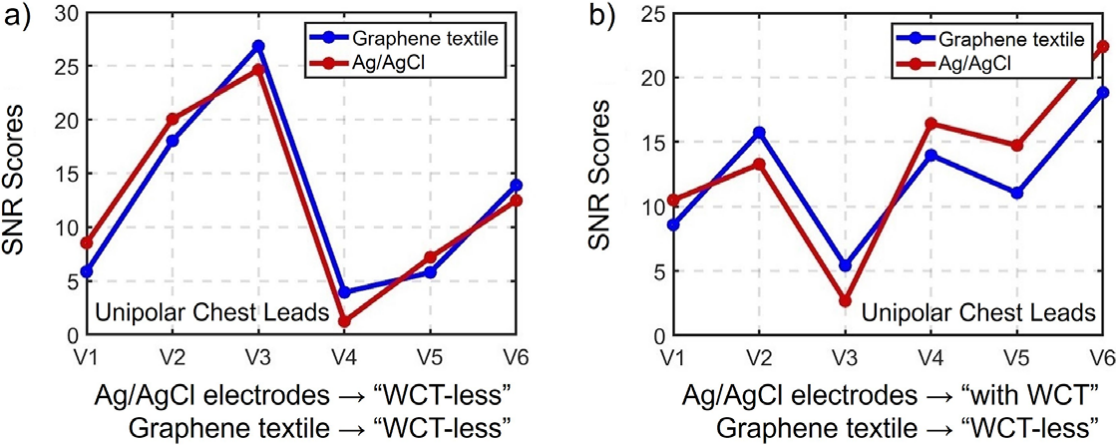
Summary of SNR (Participant #1) scores for Ag/AgCl and graphene electrodes, as detailed in Tables 1 and 2. Panel (a) shows data from experiments where both the Ag/AgCl and graphene-based armband are in “WCT-less” configuration each with 3 electrodes, while panel (b) illustrates results with WCT configuration for Ag/AgCl electrodes with a total electrode count of 10, while graphene-based armband is again in “WCT-less” configuration with a mere 3 electrodes: two on the upper arm and one on the index finger.

A more detailed signal analysis was performed and presented in Table 2. With the exception of the V1 configuration, in which the graphene textile armband exhibits a relatively low correlation ratio of %89.26 with the WCT arrangement of commercial electrodes (as previously elucidated, this lower correlation was attributed to muscle-related interference), the remaining configurations demonstrate remarkable performance, boasting a minimum correlation ratio of %95.47 and reaching a maximum of %99.54, signifying their exceptional accuracy and harmony. SNR scores also display a consistent and pleasing pattern across all leads. In lead V6, both the commercial and textile electrodes boast the highest signal quality scores of 22.4 and 18.83 dB. It is in lead V5 where the most substantial discrepancy between the two electrode types becomes evident, with a clear 3.69 dB distinction. Specifically, commercial electrodes yield an SNR of 14.73, while the armband electrodes only achieve 11.04 dB. Figure 5b shows the interrelationship between the two types of electrodes regarding their quantitative signal quality. The observation of higher RMS noise in microvolts 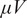 in leads V3 and V5 aligns with expectations, as the average SNR in these leads is somewhat lower compared to the others. In particular, commercial electrodes recorded the maximum RMS noise at 190.05 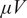 , while the graphene textile armband displayed a slightly lower value of 175.97 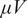 , indicating a marginal difference of only 14.08 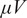 . Moreover, around the QRS complex, the mean RSS reaches its zenith, with values peaking at 5.09 mV for the armband electrodes and 4.85 mV for the commercial electrodes, particularly noticeable in lead V4. Furthermore, in lead V4, the mean RSS between QRS complexes is most prominent, measuring 1.84 mV for the armband electrodes and 1.99 mV for the commercial electrodes. This comprehensive analysis finally offers invaluable insights into the performance and variability of key parameters, including SNR, RMS, and RSS scores.

**Table 2.** Results for participant #1: Signal analysis was conducted on the graphene textile armband and compared with commercial Ag/AgCl wet electrodes that were arranged in a Wilson Central Terminal (WCT) setup

Feature 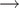 Configuration 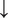		Correlation ratio	SNR score [dB]	RMS noise 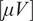	Mean RSS around QRS complexes 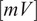	Mean RSS between QRS complexes (noise) 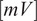
V1	Graphene textile	% 89.26	8.58	26.9	0.716	0.35
	Ag/AgCl		9.39	17.36	0.695	0.27
V2	Graphene textile	% 99.54	15.74	100.25	2.45	0.99
	Ag/AgCl		13.28	114.32	2.21	1.03
V3	Graphene textile	% 95.79	5.41	175.97	2.52	1.84
	Ag/AgCl		2.67	190.05	1.36	1.99
V4	Graphene textile	% 95.47	13.98	111.81	5.09	1.07
	Ag/AgCl		16.42	92.46	4.85	0.891
V5	Graphene textile	% 97.87	11.04	134.68	2.49	1.31
	Ag/AgCl		14.73	99.82	2.28	0.978
V6	Graphene textile	% 97.68	18.83	79.17	2.22	0.751
	Ag/AgCl		22.40	49.20	1.90	0.466

*Note.* This setup involves ten electrodes on the Ag/AgCl side (comprising six for chest leads, three for establishing the WCT, and one for reference) and three textile electrodes on the graphene armband (consisting of one differential and one reference on the upper arm, and one on the fingertip of the glove component placed on the locations of chest leads.)

The overall consistency of this signal analysis is also valid for participants 2 and 3 (available in supporting information). For the second participant, the correlation for V1 and V2 dropped to 81% and 82%, respectively, which can be attributed to biceps muscle contraction as discussed in detail above. This effect is further supported by the drop in SNR scores, as high-frequency noise contributed to the reduced correlation. However, moving to V6, the correlation for participant 2 increased to 97.72%. Likewise, for participant 3, the correlation score for V1 dropped to 72%, while V2, V5, and V6 showed correlations of 86%, 98%, and 92%, respectively. For participant 3, the lower correlation for V1 can be explained by the participant having significantly larger biceps compared to the other two participants. Nonetheless, this drop did not lead to distinct differences in signal analysis results. Signal energy remained comparable to that of commercial electrodes, and RMS noise values demonstrated strong consistency.

These observations, as a result, conclusively demonstrate that the developed armband stands as a promising and powerful tool where the wearers can now confidently utilize to record multichannel asynchronous ECG, enabling them to gain a deeper, or even diagnostic understanding of their cardiac health.

### 3.4. Discussion

Wilson terminal stands as a widely adopted reference system in nearly all medical devices in ECG measurements, but it is not without its fair share of controversies and debates. Interestingly, even among researchers working in cardiology-related fields, there appears to be no consistent understanding of what exactly is meant by the terms “central terminal,” “reference,” or “zero potential” (Bacharova et al., 2005). When the WCT was first introduced, it was conceived as an indifferent pole – a reference point created by connecting high resistances from the limb leads, where voltage variations would be so minimal as to be negligible. This allowed it to act as a near-zero potential reference for the exploratory chest electrodes (Wilson et al., 1934). However, later studies suggested that this assumption may not always hold true (Madias, 2008. This phenomenon becomes especially apparent in simulation studies, prompting the development of innovative mathematical models aimed at defining improved reference systems, including refinements of the Wilson terminal itself (Fischer et al., 2002). Some researchers have proposed front-end systems that eliminate the need for the WCT altogether, measuring potential differences without relying on the right-leg reference (Gargiulo et al., 2013a, 2013b). Others have suggested that a 9-lead configuration could provide sufficient information to replace the traditional 12-lead system (Madias, 2013). Reportedly, the accuracy of the WCT as a reference can be compromised when the electrodes are placed closer to the torso, potentially affecting the diagnostic reliability of the ECG measurements (Gargiulo, 2015). While debate continues over the necessity of the WCT, several studies have attempted to directly measure its influence on signal morphology and overall diagnostic value. For instance, one study in fetal electrocardiography found no significant advantage in using the WCT compared to direct lead comparisons (Graatsma et al., 2009). Another study replaced the WCT with single-pole right and left arm electrodes and observed minor differences – specifically, variations in ST-J amplitudes of approximately 30 to 40 microvolts (Lindow et al., 2018). Whether such differences have a meaningful impact on diagnosis remains an open question, highlighting the need for further clinical research to determine if these variations affect diagnostic accuracy for medical professionals. In the next step of our research, we aim to explore this question further by conducting a side-by-side morphological analysis, comparing signals obtained using the WCT with those recorded using our armband system that does not rely on the WCT.

One notable limitation in our study pertained to the sample size, a challenge exacerbated by the sensitive and stigmatizing nature of the subject. Finding participants willing to disrobe for the placement of chest leads and electrodes on their body proved to be a formidable task, and it would be unjust to draw hasty and confident conclusions about WCT – or the validity of our prototype – without a substantial pool of volunteers. Especially for medical-grade products, it is essential to recruit a large and diverse pool of participants to account for all potential risks, variations, and clinical usefulness. Undeterred by these limitations, our pioneering team is gearing up for a more extensive research endeavor. This forthcoming study will involve a larger participant pool within a medical-level environment, allowing us to benchmark the performance of our advanced wearable product against 12-channel commercial medical equipment commonly used in hospital intensive care units. This step aims to enhance the robustness of our findings and further validate the utility of our wearable technology. Our first step will be to recruit patients from the cardiology department and collect simultaneous ECG data using both our device and a clinical-grade ECG system. We will then compare and analyze the two datasets to assess whether our armband can accurately capture cardiac disease morphologies from a signal-processing perspective, thereby validating its clinical utility. In parallel with these clinical validation efforts, we have already initiated a national patent application in Türkiye to protect the core technology and intellectual property. We also plan to pursue patent applications in the European Union and the United States, which will support future market entry and facilitate licensing opportunities should the device move toward commercialization.

The ECG armband was designed and fabricated in a modular form as an initial prototype. This modularity allows for easy replacement of electrodes in case they become damaged – particularly since the electrodes are relatively small compared to the overall fabric area. As described in the methodology section above, each small electrode is constructed following the approach from our previous study (Ozturk et al., 2023), consisting of a metal fastener on one side and a piece of graphene-coated fabric on the other. The key component of the electrodes is the conductive textile coated with rGO. To evaluate its performance as a wearable prototype, one of the most important factors to consider is its washability, which reflects its durability. Since the conductive textile with the rGO coating is central to the wearable prototype, assessing how it degrades after repeated washing cycles would be a smart way to gauge its resilience and overall performance. In our previous empirical study, we compared two identical textile samples subjected to five washing cycles. One sample was machine-washed at 40 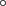 C and 400 rpm for 30 minutes, while the other was hand-washed by soaking in detergent water. The outcomes showed a clear contrast: the resistance of the hand-washed sample increased by 50%, whereas the machine-washed sample exhibited a 150% increase. This indicates that the change in resistance is not only due to the washing solution but also to the mechanical stress applied during machine washing. Therefore, hand washing is likely the better option for maintaining the durability of such wearable products over time. However, in cases where the electrodes remain intact and there is no need for washing, they can be safely stored in a closed container. Our empirical observation also shows that when kept under such conditions, there was no noticeable change in resistance for over a year. This highlights that environmental exposure alone does not significantly affect their performance – it’s the physical handling and washing that contribute most to degradation. Therefore, for long-term durability, hand washing is preferred, and if washing is unnecessary, simply keeping the electrodes in a closed, stable environment is sufficient to preserve their functionality.

Another aspect of the robustness is the interfacing of the sewn electrodes. Our empirical trials indicate that wiring plays a crucial role in suppressing motion artifacts, directly affecting the integrity of the signals generated by the armband. In this study, we demonstrated the floating electrode and touch-and-measure multilead on-demand electrocardiography in a controlled environment, whereby we utilized a nonmedical commercial electronics board, with minimal participant movement. Since unnatural motion or sudden movements are restricted in this setting, real-world performance may differ, and ongoing research worldwide continues to explore ways to enhance robustness in dynamic environments. Researchers have tested the effects of various materials for e-textile applications, such as cotton and silver yarn (Alcala-Medel et al., 2024), and have also investigated different interfacing methods (Joutsen et al., 2024). In this context, interfacing refers to how fasteners connect to different sensing components of the electrode, including various conductive fabrics, backing membranes, and other structural elements.

Moreover on robustness, discussions are also underway regarding signal filtering methods. In our current study, we employ simple, nonsophisticated offline filters. However, it is worth noting that commercial ECG devices often incorporate more intricate statistical signal-filtering techniques. This, in turn, introduces complexities surrounding the morphological characteristics of the recorded data. In our quest for scientific rigor and advancement, these topics will continue to be a focal point of our research efforts.

As graphene is a relatively new material, it has attracted significant attention over the years due to its exceptional physical, electrical, and mechanical properties. In healthcare applications, graphene has shown promising antibacterial properties, making it particularly valuable for drug delivery systems and biomedical devices (Moradi et al., 2025). Although the long-term effects of this unique material on the living are not yet fully understood and extensive studies are still ongoing to assess its full potential and safety, dermal effects of graphene, which the present study is concerned with, are reported to be minimal (Pelin et al., 2017).

As the wearables industry continues to mature, it is foreseeable that we will witness further improvements in the precision and depth of health data collected by these types of devices. Such advancements are not only beneficial for individuals seeking proactive health management but also for healthcare professionals aiming to enhance their diagnostic capabilities, potentially reducing the need for extensive hospital visits and promoting more personalized and efficient healthcare. With more accurate and comprehensive data at people’s fingertips, they can manage their health with greater confidence and make informed decisions about their well-being. This empowerment has the potential to lead to early detection of health issues and more effective self-care, ultimately enhancing the quality of life. The healthcare industry is likely to benefit significantly from these technological innovations. Medical professionals and researchers will have the chance to access to these richer, more reliable patient or disease/illness data, which can revolutionize diagnostic capabilities. In summary, the ongoing advancements in wearables promise a bright future in which individuals gain more autonomy over their health, while healthcare practitioners are equipped with an improved set of tools to deliver precise, personalized, and efficient care. This harmonious convergence of technology and healthcare will definitely transform our approach to health and well-being, impacting both individual and systemic aspects of healthcare.

## 4. Conclusion

In this study, a unique graphene textile-based armband was introduced. A distinctive feature of this armband, first time in wearables literature, is the integrated glove component, equipped with a mobile exploring electrode located at the index fingertip. This unique design empowers the wearer to position the electrode anywhere on their body within reach. While conventional textile-based wearable projects have primarily focused only on single-channel ECG measurements (i.e., lead I, II, III), the capture of multichannel ECG data is enabled by our innovative design. The development process began with the creation of a 2D design using the widely used commercial fashion design software, Clo3D. Subsequently, the design was simulated within the same software. To bring this concept to life, the armband was meticulously crafted from cotton fabric using a sewing machine. The electrodes were made using bamboo nylon textile and coated with reduced graphene, providing them with highly desirable low-resistance characteristics. The ECG capabilities of the armbands were rigorously tested against the gold standard Ag/AgCl wet electrodes’ for unipolar chest leads. These electrodes were arranged in a well-established WCT configuration, which serves as a widely recognized reference standard for all medical ECG applications.

## Supporting information

Guler et al. supplementary material Guler et al. supplementary material

## Data Availability

The dataset analyzed during this study is not publicly available but can be obtained from the corresponding author upon reasonable request.

## Supplementary material

The supplementary material for this article can be found at http://doi.org/10.1017/wtc.2025.11.
