# Supplementary material for: Wearable armband with a floating mobile exploratory electrode at fingertip for on-demand touch-and-measure multilead electrocardiography
Source: Wearable Technol. 2025 May 5;6:e21. doi: 10.1017/wtc.2025.11 (PMC12056423; doi:10.1017/wtc.2025.11)
Supplement: Guler et al. supplementary material [file S2631717625000118sup001.pdf]

Wearable Technologies (2025), **xx**:xx 1–1

CAMBRIDGE
UNIVERSITY PRESS

Supplementary Material

Wearable Armband with a Floating Mobile Exploratory Electrode at Fingertip for On-demand Touch-and-Measure Multi-lead Electrocardiography

Saygun Guler[1], Emre Aslanger[2] and Murat Kaya Yapici[1,3,4]

[1]Faculty of Engineering and Natural Sciences, Sabanci University, Tuzla, 34956, Istanbul, Türkiye,
E-mail: mkyapici@sabanciuniv.edu.
[2]Department of Cardiology, Basaksehir Pine and Sakura City Hospital, Health Sciences University, Basaksehir, 34480, Istanbul, Türkiye.
[3]Sabanci University (SUNUM) Nanotechnology Research and Application Center, Sabanci University, Tuzla, 34956, Istanbul, Türkiye.
[4]Department of Electrical and Computer Engineering, University of Washington, Seattle, 98195, Washington, USA.

1. Supplementary Data

Please refer to the next page for the signal analysis results of the second and third participants, including SNR scores and RMS noise.

	Feature → Configuration ↓	Correlation Ratio	SNR Score [dB]	RMS Noise [μV]	Mean RSS around QRS complexes [mV]	Mean RSS between QRS complexes (noise) [mV]
V1	Graphene textile	% 81.71	8.36	21.11	0.524	0.224
	Ag/AgCl		8.94	32.49	0.918	0.345
V2	Graphene textile	% 88.37	17.04	18.63	1.077	0.151
	Ag/AgCl		17.65	20.25	1.255	0.164
V3	Graphene textile	% 96.30	16.01	29.52	1.265	0.252
	Ag/AgCl		18.14	24.22	1.771	0.206
V4	Graphene textile	% 95.82	7.83	39.80	0.796	0.362
	Ag/AgCl		9.06	50.36	1.434	0.458
V5	Graphene textile	% 98.46	7.86	52.28	1.148	0.464
	Ag/AgCl		8.76	60.78	1.663	0.540
V6	Graphene textile	% 95.36	7.96	62.04	1.281	0.575
	Ag/AgCl		8.02	66.64	1.726	0.617

Table 1. *Results for participant #2: Signal analysis was conducted on the graphene textile armband and compared with commercial Ag/AgCl wet electrodes. This table shows the assessment of signal quality characteristics in the absence of a Wilson Central Terminal (WCT) setup, employing only three electrodes for each electrode type: two differentials and one reference.*

Feature → Configuration ↓		Correlation Ratio	SNR Score [dB]	RMS Noise [μV]	Mean RSS around QRS complexes [mV]	Mean RSS between QRS complexes (noise) [mV]
V1	Graphene textile	% 81.09	14.83	9.96	0.375	0.085
	Ag/AgCl		17.99	9.70	0.663	0.083
V2	Graphene textile	% 82.92	14.62	31.24	0.875	0.257
	Ag/AgCl		16.78	27.61	0.986	0.227
V3	Graphene textile	% 94.50	19.22	17.63	1.34	0.147
	Ag/AgCl		17.26	28.40	1.73	0.237
V4	Graphene textile	% 92.16	17.09	14.26	0.796	0.111
	Ag/AgCl		14.72	28.25	1.20	0.220
V5	Graphene textile	% 96.41	7.2	68.93	1.275	0.596
	Ag/AgCl		7.8	68.88	1.465	0.59
V6	Graphene textile	% 97.77	6.31	117.38	1.46	0.795
	Ag/AgCl		6.35	107.12	1.492	0.726

Table 2. *Results for participant #2: Signal analysis was conducted on the graphene textile armband and compared with commercial Ag/AgCl wet electrodes that were arranged in a Wilson Central Terminal (WCT) setup. This setup involves ten electrodes on the Ag/AgCl side (comprising six for chest leads, three for establishing the WCT, and one for reference) and three textile electrodes on the graphene armband (consisting of one differential and one reference on the upper arm, and one on the fingertip of the glove component placed on the locations of chest leads.).*

	Feature $\rightarrow$ Configuration $\downarrow$	Correlation Ratio	SNR Score [dB]	RMS Noise [μV]	Mean RSS around QRS complexes [mV]	Mean RSS between QRS complexes (noise) [mV]
V1	Graphene textile	% 81.06	6.73	45.02	1.22	0.56
	Ag/AgCl		7.01	28.73	0.822	0.361
V2	Graphene textile	% 88.0	3.23	86.16	1.496	1.029
	Ag/AgCl		-0.06	79.42	0.796	0.946
V3	Graphene textile	% 88.12	6.38	116.7	2.812	1.364
	Ag/AgCl		1.92	163.3	1.906	1.897
V4	Graphene textile	% 95.06	8.87	85.9	2.201	0.838
	Ag/AgCl		7.7	134.2	3.29	1.313
V5	Graphene textile	% 98.53	8.8	124.2	3.127	1.611
	Ag/AgCl		6.9	140.5	3.273	1.310
V6	Graphene textile	% 98.88	9.8	84.7	2.766	0.889
	Ag/AgCl		9.6	86.4	2.727	0.894

Table 3. *Results for participant #3: Signal analysis was conducted on the graphene textile armband and compared with commercial Ag/AgCl wet electrodes. This table shows the assessment of signal quality characteristics in the absence of a Wilson Central Terminal (WCT) setup, employing only three electrodes for each electrode type: two differentials and one reference.*

	Feature → Configuration ↓	Correlation Ratio	SNR Score [dB]	RMS Noise [μV]	Mean RSS around QRS complexes [mV]	Mean RSS between QRS complexes (noise) [mV]
V1	Graphene textile	% 72.01	1.35	69.31	1.02	0.874
	Ag/AgCl		9.76	49.21	1.911	0.62
V2	Graphene textile	% 86.13	3.62	132.44	1.708	1.350
	Ag/AgCl		6.07	81.707	1.67	0.833
V3	Graphene textile	% 94.56	1.16	200.67	1.695	1.760
	Ag/AgCl		2.37	191.47	1.753	1.680
V4	Graphene textile	% 91.24	11.72	44.79	1.831	0.471
	Ag/AgCl		8.06	71.47	1.914	0.752
V5	Graphene textile	% 98.16	6.57	166.2	3.254	1.527
	Ag/AgCl		6.74	126.5	2.252	1.163
V6	Graphene textile	% 92.55	8.16	142.99	3.605	1.479
	Ag/AgCl		9.6	63.16	1.981	0.789

Table 4. *Results for participant #3: Signal analysis was conducted on the graphene textile armband and compared with commercial Ag/AgCl wet electrodes that were arranged in a Wilson Central Terminal (WCT) setup. This setup involves ten electrodes on the Ag/AgCl side (comprising six for chest leads, three for establishing the WCT, and one for reference) and three textile electrodes on the graphene armband (consisting of one differential and one reference on the upper arm, and one on the fingertip of the glove component placed on the locations of chest leads.).*

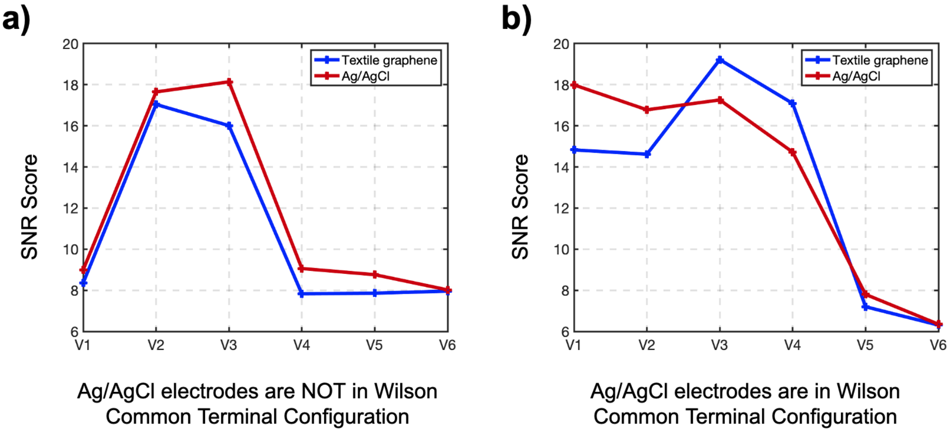

Figure 1. *Summary of SNR (Participant #2) scores for Ag/AgCl and graphene electrodes, as detailed in Tables above. Panel (a) shows data from experiments where both the Ag/AgCl and graphene-based armband are in "WCT-less" configuration each with 3 electrodes, while panel (b) illustrates results with WCT configuration for Ag/AgCl electrodes with a total electrode count of 10, while graphene-based armband is again in "WCT-less" configuration with a mere 3 electrodes: two on the upper arm and one on the index finger..*

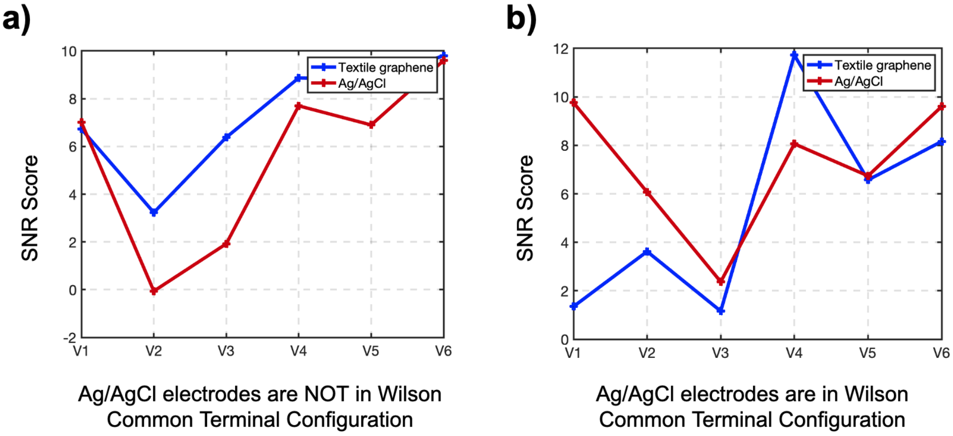

Figure 2. *Summary of SNR (Participant #3) scores for Ag/AgCl and graphene electrodes, as detailed in Tables above. Panel (a) shows data from experiments where both the Ag/AgCl and graphene-based armband are in "WCT-less" configuration each with 3 electrodes, while panel (b) illustrates results with WCT configuration for Ag/AgCl electrodes with a total electrode count of 10, while graphene-based armband is again in "WCT-less" configuration with a mere 3 electrodes: two on the upper arm and one on the index finger..*
